# Supplementary material for: Stage-Specific Effects of TiO2, ZnO, and CuO Nanoparticles on Green Microalga Haematococcus lacustris: Biomass and Astaxanthin Biosynthesis
Source: Mar Drugs. 2025 May 11;23(5):204. doi: 10.3390/md23050204 (PMC12113255; doi:10.3390/md23050204)
Supplement: Supplementary file 1 [file marinedrugs-23-00204-s001.zip › marinedrugs-3579796-SM.pdf]

Table S1. Pearson correlation coefficients (r) between nanoparticle concentrations and biochemical parameters, and among the analyzed parameters in *Haematococcus lacustris*.

Nanoparticles	*Haematococcus lacustris*, life cycle stages		
	Green motile cells	Palmella	Aplanospore
	Correlation (r): NPs concentration (mgL) – biomass content (g/L)		
TiO_2	-0.24901	-0.32228	0.784425
ZnO	0.74317	0.780019	0.269096
CuO	0.44674	0.63306	-0.84756
	Correlation (r): NPs concentration (mg/L) – astaxanthin content (% biomass)		
TiO_2		-0.86515	0.551753
ZnO		0.900082	-0.91209
CuO		0.183393	-0.84941
	Correlation (r): NPs concentration (mg/L) – lipids content (% biomass)		
TiO_2		0.540075	0.43272
ZnO		-0.82730	-0.81912
CuO		-0.32104	-0.81037
	Correlation (r): lipids content (% biomass) – astaxanthin content (% biomass)		
TiO2		-0.46582	0.965579
ZnO		0.94110	0.820152
CuO		-0.12014	0.902022
